# Episodic future thinking modulates delay discounting in individuals with problematic substance use: a narrative review

**DOI:** 10.3389/fpsyt.2026.1760734

**Published:** 2026-02-02

**Authors:** Xiwen Chen, Huanxin Wang, Cong Fan

**Affiliations:** 1 Institute of Psychological and Brain Sciences, Liaoning Normal University, Dalian, China; 2 Key Laboratory of Brain and Cognitive Neuroscience, Dalian, Liaoning, China

**Keywords:** delay discounting, episodic future thinking, intervention, regulatory mechanisms, substance use disorder

## Abstract

Substance use disorder (SUD) poses significant challenges to public health, the economy, and social safety. Delay discounting (DD) is one form of impulsivity which is a risk factor for SUD and other mental health disorders. Moreover, when faced with immediate rewards, individuals with SUD exhibit increased DD compared to healthy controls. Fortunately, previous studies have shown that EFT, referring to vividly imagining potential future events in specific scenarios based on an individual’s current experiences, can effectively reduce DD and substance use in individuals with SUD. In this process, the potential regulatory mechanism of EFT may involve extending the temporal window and decreasing the construal level of future events. Most promising avenues to pursue in future studies may include manipulating participants’ factors (e.g., sample size, adherence to diagnostic criteria, comorbidity with other mental diseases), conducting longitudinal studies and exploring the neural regulatory mechanisms of EFT on DD among individuals with SUD. Further research should also examine the effects of both positive and negative emotional valence in EFT interventions to determine how different types of future thinking influence impulsivity and decision-making. This would help us develop more applicable theoretical models and improve the effectiveness of EFT in intervening with SUD.

## 1 Introduction

Substance use disorder (SUD) refers to addictive mental disorders caused by the use of psychoactive substances, including substance abuse and substance dependence (1). The substances involve alcohol, opioids, cannabis, nicotine, cocaine, and so on (2). SUD damages not only individuals’ physiological systems, such as respiration, endocrine, and cardiovascular functions, but also their cognitive functions (3). Moreover, it imposes a significant burden on social safety and national finances (4). Given the multifaceted burden of SUD, it is crucial to prioritize effective therapeutic strategies. Therefore, the current research on SUD interventions aims to uncover effective approaches to reduce substance consumption and guide therapeutic practices (5).

Impulsivity, an underlying vulnerability marker for SUD (6), is characterized as a maladaptive inhibitory process (7). Frequently, researchers measure impulsivity in individuals with SUD utilizing delay discounting (DD) tasks, which assess the tendency to devalue rewards as the delay in obtaining them increases (8). They have found that compared to healthy controls, people with SUD tend to make more impulsive choices in intertemporal decision-making processes, preferring immediate but smaller rewards over delayed but larger rewards. The findings reveal that individuals with SUD exhibit higher DD rates, indicating greater impulsivity (9–12). Therefore, reducing impulsivity could be regarded as a breakthrough in elucidating the relationship between DD and SUD. By investigating the underlying processing mechanisms, we can help individuals with SUD make more long-term and healthier decisions by inhibiting their impulsivity, ultimately leading to more effective interventions.

Episodic future thinking (EFT) is one of the effective interventions for improving SUD (13). EFT is an effective method for reducing DD as it decreases impulsivity and suppresses the demand for substances (14). Building on existing reviews that primarily focus on general intervention outcomes (15, 16), our review aimed to confirm the feasibility of EFT in reducing impulsivity in individuals with SUD by summarizing the evidence for the effects of DD on substance use and the effects of EFT for reducing DD and substance use with the aim of then proposing potential mechanisms that may mediate the effects of EFT.

## 2 DD in individuals with SUD

In daily life, decisions often involve balancing the present and the future. For example, should I withdraw money from the bank now or several years later? Should I indulge in pleasures now or save money for retirement? Should I take drugs at this moment or pursue a healthier lifestyle in the future? During this process, as the delay time increases, individuals tend to devalue or discount future outcomes, a phenomenon known as DD. DD is commonly represented by a hyperbolic discounting function (SVLL=A/(1+k*D)), in which SVLL represents the subjective value of a large but long-delayed reward, A represents the amount of reward obtained after a particular delay, D represents the delay time, and k represents the discount rate (17). A steeper discounting curve indicates a stronger preference for immediate but smaller rewards (18).

Individuals with SUD commonly prefer immediate substance rewards over long-term health gains, making DD an effective measure of impulsive decision-making and a pathological feature of SUD (8). Behavioral studies have found significantly greater DD rates in individuals with current alcohol use disorder compared to healthy individuals, as well as higher rates in untreated alcohol use disorder individuals compared to those seeking treatment (19). Furthermore, similar findings have been observed in individuals with other SUDs, such as nicotine and opioid use disorders, indicating increased impulsivity in populations with SUD (9, 10).

Although elevated DD is one of the most consistently observed behavioral correlates of SUD, its causal status remains actively debated. A growing body of work has questioned whether DD should be conceptualized as a core or primary pathological mechanism of addiction, highlighting concerns regarding construct validity, limited cross-task generalizability, and weak specificity to particular forms of psychopathology (20, 21). From this perspective, steeper DD, which captures individual differences in impulsive choice and future-oriented valuation, may reflect a broader dimension of impulsive or uncertain decision-making rather than a unitary causal driver of substance use behavior. Nevertheless, elevated DD is one of the most robust and consistently observed behavioral characteristics across substance-using populations and has been reliably associated with substance use severity, craving, and impulsive decision-making across a wide range of substances (11, 22, 23).

Furthermore, empirical evidence suggests that the relationship between DD and SUD is heterogeneous and potentially bidirectional. On the one hand, longitudinal and high-risk cohort studies indicate that elevated DD can precede the onset and escalation of substance use, particularly among individuals exposed to early-life adversity or familial risk for SUD (24–26). Higher DD in adolescence has been shown to predict subsequent increases in substance use and related problems, supporting its role as a vulnerability marker or intermediate phenotype. On the other hand, evidence that substance use produces enduring increases in DD is more limited, with most findings pointing to transient, state-dependent effects associated with acute intoxication, withdrawal, or contextual stressors rather than stable trait-level change (27, 28). Together, these findings argue against a simple causal interpretation while underscoring the relevance of DD as a behaviorally meaningful dimension associated with substance use risk.

In line with the aforementioned debate, this review does not regard DD as a comprehensive index of addiction. Instead, DD is emphasized due to its functional role as a sensitive and theoretically interpretable behavioral index in the context of EFT–based interventions. EFT is specifically designed to engage future-oriented cognitive processes, and DD reflects changes in how delayed rewards are valued vs. immediate rewards. Empirical studies consistently demonstrate that EFT produces reliable reductions in DD across substance-using and at-risk populations (14, 29), likely by extending individuals’ effective temporal window and increasing the subjective value of future outcomes. In this sense, DD may not serve as a single index of clinical interest per se, but as a tractable behavioral dimension through which EFT-related cognitive change can be detected.

Importantly, reductions in DD should not be interpreted as a guaranteed or sufficient mediator of substance use change. Although longitudinal evidence indicates that elevated DD prospectively predicts substance use escalation and statistically mediates associations between early environmental risk factors (e.g., socioeconomic disadvantage, parental psychopathology) and later substance use outcomes (30; 26), this developmental mediation framework does not directly generalize to intervention contexts. In the context of EFT, existing studies frequently demonstrate parallel reductions in DD and substance-related outcomes, such as decreased substance demand or real-world consumption (31, 32). However, relatively few studies have employed formal mediation analyses or longitudinal designs capable of establishing temporal precedence between EFT-induced changes in DD and subsequent changes in substance use behavior. Moreover, emerging evidence suggests that EFT can reduce substance use outcomes even in the absence of reliable changes in DD, particularly when EFT cues are domain-specific or explicitly linked to substance-related goals (33).

Accordingly, the present review treats DD not as a definitive causal driver of SUD, but as a theoretically informative and experimentally tractable outcome that captures one pathway through which EFT may exert its effects in SUD individuals. At the same time, we acknowledge important limitations: DD reflects only a narrow facet of impulsive choice, may be influenced by environmental uncertainty rather than impulsivity per se, and lacks disorder specificity (21, 25). A balanced interpretation therefore, requires situating DD within a broader network of cognitive, affective, and contextual processes that jointly shape substance use behavior.

## 3 The intervention of EFT on DD in individuals with SUD

**Figure 1:**
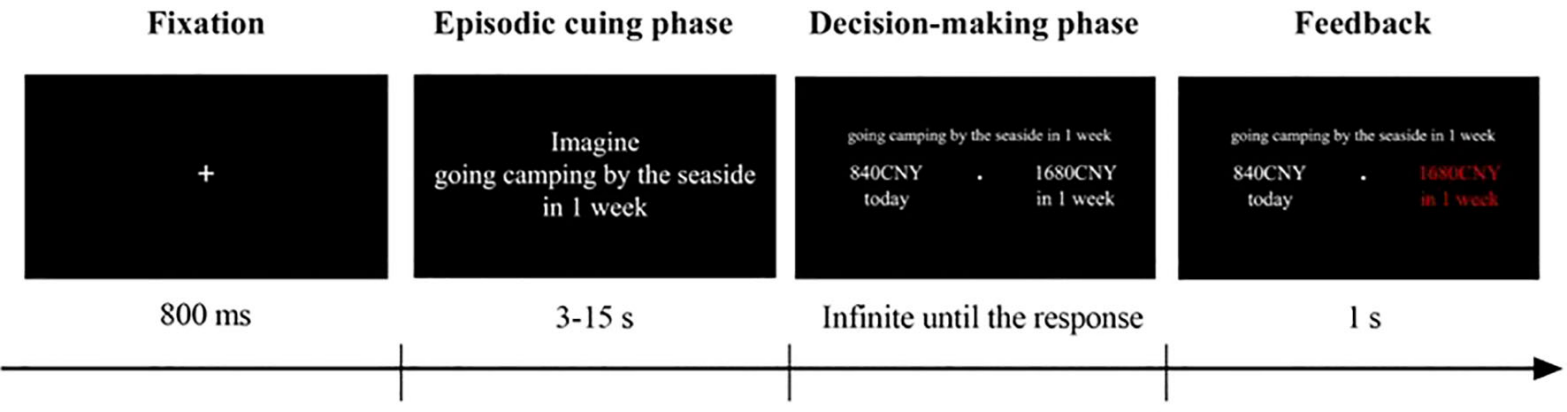
The procedure of the cued DD task (35): At the beginning of each trial, a fixation was presented for 800 ms. Then, an episodic event with one delayed time was displayed, and participants needed to imagine the future experience as vividly as possible in 15 s. After imaging for at least 3 s, they were allowed to press the space bar to enter the decision phase. They then entered the decision-making phase and made a decision between a smaller but immediate reward and a larger but delayed reward without the time limitation. Finally, feedback on their choice was provided for 1 s.

EFT involves the mental simulation of future events and typically requires two abilities: vividly constructing detailed scenarios of future events and engaging in “mental time travel” (34). Evidence reveals that people frequently imagine positive and desirable futures, which makes the future more concrete. This process promotes intertemporal decision-making by considering the immediate and long-term outcomes of their actions (14). Participants in EFT experimental tasks are asked to imagine events (e.g., a planned holiday or time spent with family) at future time points, such as one day, one week, or one month and then incorporate event-related cues into DD tasks (see **Figure 1**).

### 3.1 Mechanistic relevance of EFT for SUD

EFT may be particularly relevant for individuals with SUD because it is mechanistically well aligned with the cognitive–motivational impairments that are characteristic of SUD. Individuals with addictive disorders consistently exhibit a markedly shortened temporal window, reflecting a reduced capacity to project themselves into the future and to integrate delayed consequences into present decision-making (23, 36). This deficit directly undermines the effectiveness of many standard addiction treatments that rely on abstract, future-oriented reasoning (e.g., long-term health benefits of abstinence). EFT is uniquely positioned to target this impairment by transforming distal outcomes into vivid, personally relevant episodic representations, thereby increasing the psychological value and immediacy of future rewards (37, 38). Meta-analytic evidence further indicates that EFT produces moderate and robust reductions in DD, with effect sizes comparable to or exceeding many alternative DD-focused interventions (39, 40). EFT is by no means the only intervention shown to reduce DD. The emphasis on EFT, however, rests on the fact that its core mechanism—episodic prospection—directly addresses a central cognitive vulnerability in substance use disorders: an impaired capacity to effectively represent and assign value to one’s future self and outcomes (41). Consequently, EFT should be conceptualized not merely as a strategy for manipulating DD, but as an intervention with particular theoretical and clinical relevance for addictive disorders.

### 3.2 Empirical evidence for EFT interventions on DD in SUD

EFT could effectively reduce impulsivity in individuals with SUD during DD tasks (see **Table 1**). For instance, in an investigation into the intervention effects of EFT on cocaine use disorder, researchers interviewed eighteen cocaine users about the simulation of future events before the task. Subsequently, they added these future events into the DD task to compare the differences between the control (DD task without future event cues) and EFT conditions. The results showed that, relative to the control condition, EFT reduced the discounting of future rewards in individuals with SUD (50). EFT has significant intervention potential, as evidenced by its consistent success in nicotine, cannabis, alcohol, and heroin use disorders (19, 43, 49). Several studies have also incorporated negative consequences of substance use and individuals’ health goals into imagined scenarios, demonstrating that scenarios involving negative consequences and health goals were more effective in reducing demand for a substance than general content scenarios (42, 54, 57). Nonetheless, when compared to general content scenarios, scenarios regarding undesirable consequences and health goals did not reduce DD (57), but instead increased DD (42), indicating that scenarios about the negative consequences of substance use may potentially worsen impulsive behaviors. Additionally, the negative emotions triggered by adverse consequences may diminish compliance with this intervention, thereby careful consideration is needed when selecting the content for EFT.

**Table 1:** Studies regarding EFT effect on DD in SUD individuals.

Reference	Substance type	Sample size	Type of EFT	EFT effect on DD	Substance demand/intake	Substance craving
(42)	cigarette	50	positive	decrease	decrease	decrease
(43)	cigarette	29	positive	NS	decrease	--
(44)	cigarette	72	positive	decrease	decrease	--
(45)	cigarette	189	positive	decrease	decrease	decrease
(46)	cigarette	117	positive	decrease	decrease	NS
(47)	cigarette	90	positive	decrease	decrease	--
(31)	cigarette	54	positive	decrease	decrease	--
(48)	cigarette	210	positive	NS	NS	--
(49)	cannabis	200	positive	decrease	--	--
(33)	cannabis	90	positive	NS	decrease	--
(50)	cocaine	18	positive/ neutral	decrease	--	--
(51)	cocaine	44	positive	decrease	decrease	--
(52)	alcohol	48	positive	decrease	decrease	--
(38)	alcohol	50	positive	decrease	decrease	--
(53)	alcohol	55	positive	decrease	decrease	--
(54)	alcohol	137	positive	decrease	NS	--
(54)	alcohol	138	negative	decrease	NS	--
(55)	alcohol	50	positive	decrease	--	--
(32)	alcohol	52	positive	decrease	decrease	--
(56)	alcohol	45	positive	NS	decrease	--
(13)	alcohol	28	positive	decrease	decrease	NS

NS means no significant; -- means the study did not measure the index.

Aside from reducing DD, in clinical trials involving individuals with SUD, EFT is often used as a novel intervention to reduce substance demand. In addition to administering DD tasks, hypothetical purchase tasks and questionnaires on substance demand or dependence are used as supplemental explanations to examine changes in participants’ demand for substances (43, 46, 49). For example, Stein et al. found that EFT effectively reduced DD and decreased demand for cigarettes in people with nicotine use disorder during hypothetical purchase tasks (46). Consequently, we propose that reduced DD may be associated with decreased substance demand.

Moreover, EFT has been extended from laboratory tasks to real-world applications. By investigating its effects on real-life alcohol consumption, Athamneh et al. (32) proved its effectiveness in reducing demand for alcohol in individuals with alcohol use disorder. The study was divided into two phases: baseline and intervention, with laboratory and real-life components in each. During the baseline phase, participants completed demographic information, assessments (such as the Beck Depression Inventory and Beck Anxiety Inventory, Alcohol Use Disorders Identification Test, and alcohol craving questionnaire), and the DD task. During the real-life phase, they reported their alcohol consumption daily. Following the baseline phase, eligible participants continued to the intervention phase, which also had laboratory and real-life components. During the laboratory intervention phase, participants were instructed to engage in EFT by imagining and describing future events, as well as rating their imagined scenarios. During the real-life intervention phase, researchers recorded their alcohol use for 14 days and collected breath samples daily. Then, participants were reminded to imagine EFT events twice a day via text message. The study found that remote EFT intervention significantly reduced daily alcohol drinking. However, another study found a lower adherence rate (low compliance) (43), suggesting the need for EFT implementation improvements, such as shorter intervals, fewer practice sessions, and increased monitoring to enhance its feasibility and effectiveness in real-world settings. These changes are crucial for maximizing the intervention’s impact and ensuring its successful implementation into clinical practice.

In summary, EFT is an emerging and promising intervention strategy that reduces both impulsivity and substance cravings in individuals with SUD (32; 54). This is supported by empirical evidence, which has shown positive effects in real-world contexts, demonstrating a certain ecological validity. Understanding the underlying mechanisms of these effects will provide theoretical support and open new avenues for future investigation. Therefore, we intend to review the potential mechanisms underlying the impact of EFT on DD in people who suffer from SUD.

## 4 The potential regulatory mechanism of EFT on DD in individuals with SUD

**Figure 2:**
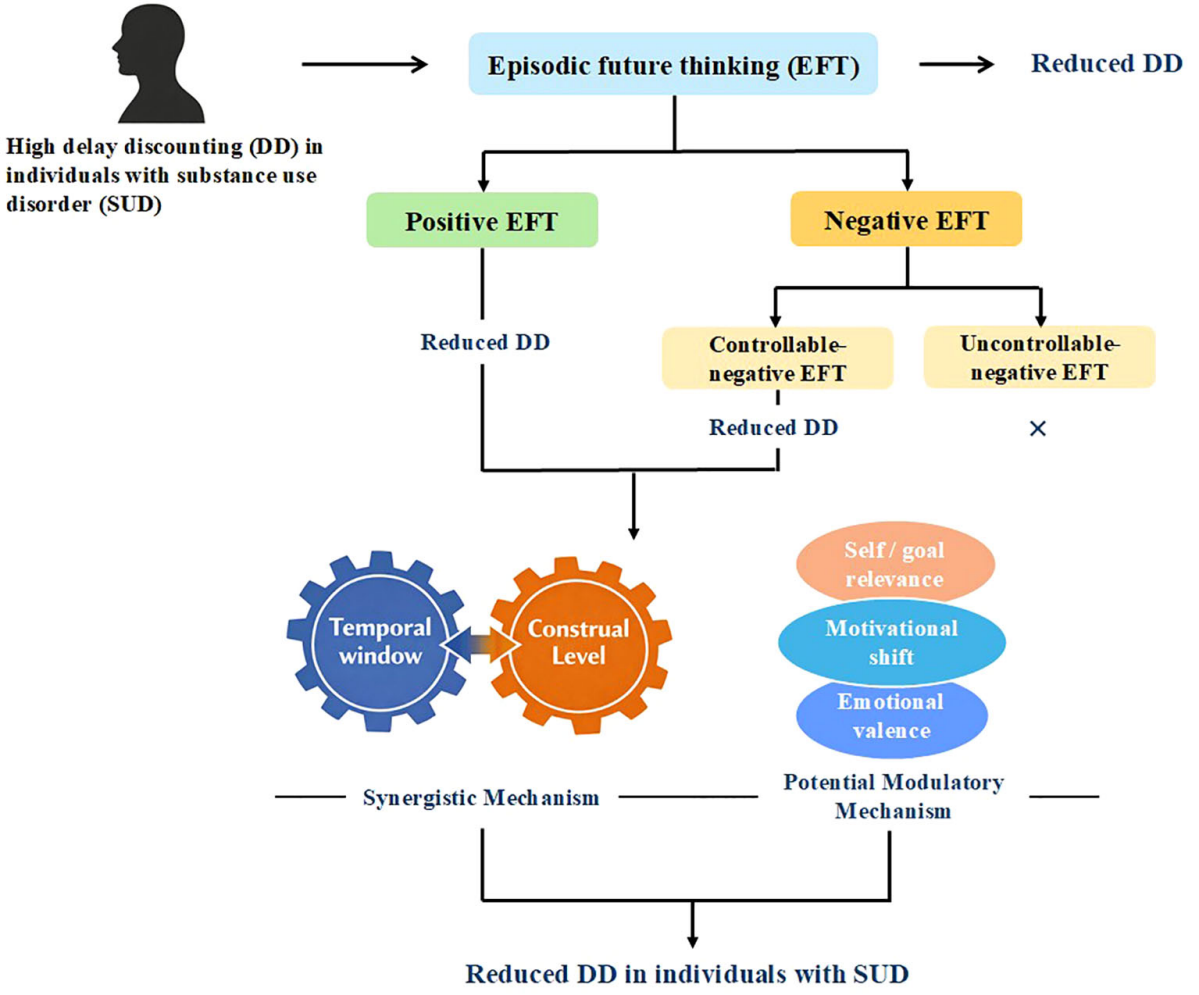
Conceptual model of how EFT modulates DD in individuals with SUD. Individuals with SUD typically exhibit elevated levels of delay discounting, reflecting a strong preference for immediate rewards. EFT is proposed to reduce DD, with positive EFT producing robust reductions. Within negative EFT, outcomes depend on perceived controllability: controllable negative EFT is associated with reduced DD, whereas uncontrollable negative EFT shows little or no effect. These effects are theoretically grounded in a unified cognitive framework in which Temporal Window and Construal Level Theory represent functionally distinct yet mutually dependent mechanisms. Additionally, self-/goal-relevance, affective engagement, and motivational shifts are potential modulatory mechanisms that influence the magnitude and durability of EFT effects. Ultimately, these mechanisms promoting greater self-control reduce DD in individuals with SUD.

Several modulatory mechanisms may explain how EFT affects DD in individuals with SUD. A conceptual figure illustrating how the proposed mechanisms, including temporal window expansion, changes in construal level, and other potentially relevant processes, are assumed to relate to each other and jointly influence DD (see **Figure 2**).

### 4.1 The extended temporal window by EFT where individuals with SUD integrate the value of substance

The temporal window may be shortened in individuals with SUD. The temporal window, defined as an individual’s imagined future time distance (36), indicates how people organize their behavior to achieve future goals (58). The Reinforcer Pathology Theory 2.0 elucidates the synergistic relationship between temporal discounting processes and behavioral demand mechanisms by conceptualizing how individuals connect the valuation of rewards to their anticipated temporal contexts. Based on the theory, an excessive preference for substance reinforcers results from narrow temporal window (i.e., brief, intense, immediate, and reliable) (23). Early studies examined whether the temporal window of individuals with heroin was shortened. Healthy individuals and individuals with heroin were asked to list ten events that would happen in their lives, and after each event was described, the subjects were required when the event was most likely to occur. The results revealed that healthy individuals reported an average time range of about 5 years for imagining the future, whereas individuals with heroin reported an average time range of about 9 days (36), indicating that individuals with heroin have shorter temporal windows.

Additionally, individuals with SUD and a shorter temporal window are associated with higher DD. Individuals with SUD who were assessed using the Zimbardo Time Perspective Inventory were found to focus more on present pleasure (present hedonism) and plan less for the future, with the severity of SUD symptoms and DD rate being positively correlated with present-fatalistic scores and negatively correlated with the future scores (58). Additionally, DD measures the temporal window (i.e., how far the individual can imagine into the future) available for reinforcer value integration (59). These findings indicate that high DD rates correlate with a perceived lack of control over the future and inadequate future preparation. Taken together, individuals with SUD and a shorter temporal window are connected with higher DD.

Notably, EFT may regulate DD in individuals with SUD by extending their temporal window (15). According to social cognitive and goal-based motivation theory, people’s viewpoints on the future are fundamental determinants of behavior (60), particularly in goal-setting and self-regulation. Future time perspective refers to the current expectations of near or distant future goals. Specifically, individuals with shorter future time perspectives develop shorter-term goals, whereas those with longer future time perspectives set longer-term goals (61). Furthermore, individuals having a longer time perspective decline in the subjective value of the DD function less than those with a shorter time perspective. Therefore, they are thought to be more capable of accurately predicting the consequences of their behaviors and identifying the connection between current behaviors and future benefits (61). Importantly, based on Reinforcer Pathology Theory 2.0, the method to extend the temporal window can decrease the value for substance abuse (23). In SUD research, adding future events related to long-term health or career goals increased the future temporal window for individuals with SUD, reducing DD during decision-making and showing positive intervention effects (45, 50). Consequently, EFT may help SUD individuals better ascribe higher incentive value to future goals by extending the temporal window, resulting in more delayed choices in DD tasks.

### 4.2 The decrease in construal level of future events

According to Construal Level Theory (CLT), there is a systematic difference in the abstractness level of descriptions of near and distant future events. When describing near-future events, people tend to use more specific and detailed statements (low construal level), whereas when describing distant future events, they tend to use more abstract and decontextualized statements (high construal level) (62). Additionally, CLT posits that the increase of psychological distance leads to abstract (high) construal (62). EFT may reduce perceived temporal distance by rendering future outcomes more vivid and concrete (low construal) (63), reflecting a shift from abstract to concrete representations of future events. Taken together, EFT may counteract the natural tendency of SUD individuals to view the future abstractly, thereby increasing the subjective value of delayed rewards.

Exerting a low construal level in the future can inhibit impulsive behavior. Yi et al. explored the influence of construal level on DD (64). In study 2, some participants were required to complete DD tasks while answering questions designed to prime concrete construal of the future or the present. In contrast, other participants were instructed to complete DD tasks while answering questions designed to prime abstract construal of the future or the present. The researchers found that low construal of the future could increase the preference for delay rewards. Meanwhile, abstract construction of present events could lessen the preference for immediate results, thereby reducing DD (64). The result was in line with Kim’s study (65). The finding can be attributed to the different psychological processes involved in addressing immediate reward versus delayed reward. For example, according to CTL, delayed rewards are psychologically more distant, and therefore, tend to be more abstractly constructed than immediate rewards (62). Thus, lowering the construal level of the future may enhance the appeal of delayed rewards.

Low construal levels, which are often associated with vivid and detailed mental representations, have a major impact on substance use-related behaviors. According to one study on opioid use, concrete construal level can reduce opioid use and is linked to increased specificity and perceived urgency regarding the negative consequences of substance abuse (66). On the one hand, health messages conveyed in concrete language improve people’s perceptions of substance abuse risks, potentially resulting in fewer intents to use substances. On the other hand, a lower level of construal is associated with a decreased perceived temporal distance, which may correspond to a reduction in uncertainty regarding the considered events (62). Based on the theory of environmental controllability, uncertainty about the environment affects people’s long-term investment and decision-making behaviors (67). Consequently, the reduction in uncertainty induced by low construal level biases individuals toward choosing delayed rewards. Furthermore, in the study by Maurer Herter et al., low construal levels increased the effectiveness of rational health messaging in promoting smoking cessation and reducing alcohol consumption (68). This concrete method of thinking helps individuals with SUD develop and pursue self-improvement goals, which promotes healthier behavior modification.

However, given that all the EFT events currently used are mainly positive, it is unclear whether the regulatory mechanism will be applied to negative EFT events. If someone elaborates more on the negative outcomes in the future, people are less willing to wait for these results. Future research can involve an in-depth exploration and comparison of regulatory mechanisms for different emotional valences to confirm whether the explanation of this potential regulatory mechanism holds true for both positive and negative EFT on modulating DD.

Although we discuss “temporal window expansion” and “construal-level shifts” as two candidate mechanisms, they are not independent pathways but mutually modulate DD induced by EFT. In Reinforcer Pathology Theory 2.0, DD indexes the temporal window over which reinforcer value is integrated; high discounting reflects a narrowed temporal window that prioritizes brief, immediate, and reliable reinforcers (23, 59). Construal Level Theory, in contrast, specifies how future outcomes are mentally represented: psychologically distal events tend to be construed more abstractly, whereas psychologically proximal events are represented more concretely; and importantly, the relationship between psychological distance and construal level is bidirectional (62; 69). From this perspective, EFT expands the temporal window by changing the format of future representations: vividly simulating episodic details shifts distant outcomes toward a more concrete construal, which reduces perceived temporal distance and uncertainty and makes future consequences more cognitively accessible at the time of choice. Consistent with this account, manipulating representations to make the construal of present and future options more similar (e.g., concretizing the future) reduces DD (65). Thus, EFT can be viewed as a single intervention that simultaneously (i) broadens the horizon over which outcomes are integrated and (ii) “brings” distal outcomes psychologically closer via concretization; together, these changes increase the subjective weight of delayed rewards and thereby reduce DD. As a result, individuals with SUD may find it easier to make healthier decisions that prioritize long-term goals over immediate gratification, reducing impulsivity and substance demand.

### 4.3 Other general psychological processes

In addition to the two mechanisms emphasized above, EFT inevitably recruits more general cognitive–affective processes that may contribute to, or moderate, its effects in SUD. First, EFT cues are typically autobiographical and goal-relevant. By increasing the personal relevance of future events and activating personally meaningful goals, EFT may make delayed outcomes more cognitively accessible at the time of decision-making, thereby increasing the likelihood that these future consequences are taken into account in their choices. (38, 70, 71). Second, EFT may operate as a motivational ‘amplifier’ by making valued future activities more vivid and actionable, thereby strengthening commitment to long-term goals (i.e., reducing DD) and weakening the momentary motivational advantage of drug reinforcement (31, 72). Third, EFT reliably elicits affective and prospective emotional responses, including increased enjoyment, excitement, and emotional engagement with future events (31, 38, 73). Rather than operating as a simple “positive mood” mechanism, emotion appears to support EFT by enhancing engagement with future simulations and increasing the vividness and goal relevance of delayed outcomes, thereby facilitating their consideration during intertemporal choice (38, 73; 63, 74), which suggests that these processes can amplify each other’s effects. Therefore, self-relevance/goal relevance, motivational shifts, and affective engagement may be conceptualized as interacting processes that work synergistically to enhance the effects of EFT on DD in individuals with SUD.

Taken together, in addition to the extended temporal window and decreased construal level as the primary regulatory variables of EFT’s impact on DD, self-relevance/goal relevance, motivational shifts, and affective engagement may be best viewed as interacting processes that can enhance (or, in some contexts, limit) the magnitude and durability of EFT effects in SUD.

## 5 Conclusion and future directions

Following a review of the aforementioned studies, DD has been identified as an effective behavioral indicator of impulsivity in individuals with SUD. SUD is a risk factor for DD, and a thorough understanding of DD can provide insights into SUD intervention and treatment strategies. EFT may effectively reduce DD in individuals with SUD by extending their time window and decreasing their representation of future events, thus inhibiting impulsivity and yielding positive intervention outcomes. However, several issues in this field require further exploration.

Firstly, prior studies had several limitations in terms of participant sampling. Some studies used small sample sizes, which could result in insufficient statistical power to detect significant differences (42, 43). Individuals with SUD may also have comorbidity with other disorders (75), and most studies have not comprehensively considered psychopathology, such as personality disorders, eating disorders, or other mental disorders. Additionally, the severity of symptoms in participants who have not undergone formal diagnostic screening is lower than that of clinical samples that meet diagnostic criteria, and their performance on DD tasks does not significantly differ from that of healthy individuals (75). Future research should consider larger sample sizes from diverse populations to ensure the generalization of the findings. It is also important to thoroughly assess other characteristics of individuals with SUD, such as demographic factors, particularly comorbid disorders. Higher DD may reflect various underlying mechanisms, such as decreased executive control, increased reward processing, or a sense of hopelessness about the future. Exploring the potential different behavioral or neurobiological mechanisms and determining the relationships among these factors may be an interesting topic for future research. Furthermore, using strict diagnostic criteria to screen participants (e.g., the Diagnostic and Statistical Manual of Mental Disorders, Fifth Edition, DSM-5) can improve the generalizability of intervention measures.

Secondly, future research should move beyond reliance on DD as a single outcome index and adopt multidimensional assessment frameworks when evaluating EFT-based interventions in SUD. Although DD provides a theoretically central and empirically sensitive dimension of change, substance use behavior is influenced by multiple interacting processes. Complementary indices—including substance demand elasticity, real-world consumption metrics, craving dynamics, executive control measures, and temporal window assessments—should be integrated to more comprehensively characterize intervention effects (23, 32, 76). Such integrative approaches may clarify when changes in DD translate into meaningful clinical outcomes and help identify which combinations of behavioral indices are most informative for monitoring treatment response and tailoring EFT-based interventions. In this sense, DD should be regarded as a necessary but not sufficient dimension within a broader set of indices for understanding and optimizing EFT-related behavior change in SUD.

Thirdly, long-term track and record of the regulatory effects of EFT on DD in individuals with SUD warrants further investigation. Recovery from SUD is a dynamic process with the risk of relapse (77). Most previous studies that are cross-sectional have explored the relationship between perceived risk of relapse and DD, as well as the short-term regulatory effects of EFT on DD in individuals with SUD (43, 49, 50). Nevertheless, the long-term linkage between DD and fluctuations in relapse remains unclear. Moreover, in studies on the regulatory effects of EFT on DD, participants were only assessed with DD tasks before and after EFT intervention, which may not have captured potential DD changes. Before implementing EFT in clinical treatment, it is necessary to lengthen the intervention period of EFT and regularly measure the performance of individuals with SUD on DD tasks. Describing the long-term trajectory of recovery and the effects of EFT by examining DD changes and the variations in relapse or remission stages may provide valuable insights.

Fourthly, methodological heterogeneity represents an important limitation in the EFT effect on DD in SUD. Existing EFT-related studies vary substantially in key procedural features, including the temporal distance of imagined events, emotional valence, degree of self- and goal-relevance, and frequency or duration of EFT practice (16, 39, 71). Because these dimensions are often manipulated simultaneously or inconsistently across studies, it remains difficult to determine which specific components are necessary or sufficient for reducing DD or substance-related outcomes. Therefore, EFT should not be regarded as a unitary or standardized intervention, and effect sizes observed across studies likely reflect interactions among multiple design features rather than a single underlying mechanism. Future research should explicitly address this heterogeneity by adopting more systematic and component-based experimental designs (e.g., independently manipulate core dimensions such as temporal distance, emotional valence, and goal relevance within unified paradigms), ultimately improving the translational potential of EFT-based interventions on DD in SUD individuals.

Fifthly, negative-valence EFT may be as beneficial as positive-valence EFT to reduce DD for individuals with SUD. Previous studies have often adopted positive events as the material for EFT events because of the stability and effectiveness of positive-valence EFT in reducing DD (45, 50, 57, 78). Conversely, several studies have shown that imagining the negative outcomes leads to greater impulsivity and a stronger preference for immediate rewards by inducing stress or avoidance behaviors (63, 74, 79). Importantly, emerging evidence suggests that negative-valence EFT is not uniformly maladaptive. We highlighted the potential of negative-controllable EFT, which involves imagining negative future events that the individual can control, such as coping with future life challenges or managing stress (35). These types of negative future scenarios promote positive emotions and drive individuals to regulate themselves (80) and strive toward achieving goals (81). Therefore, while “scare tactics” based on substance use consequences may be detrimental, simulating controllable negative life events may enhance coping strategies, reduce impulsivity, and increase the value placed on delayed rewards. To avoid confusion in clinical applications, we recommend that future interventions using negative-valence EFT carefully differentiate between these two types of negative thinking, ultimately promoting better decision-making and long-term behavior change in individuals with SUD.

Sixthly, comorbidities are a significant challenge when treating individuals with SUDs as the majority of them frequently meet diagnostic criteria for two or more disorders simultaneously. Major depressive disorder (MDD) is frequently comorbid with SUDs, particularly alcohol use disorder, and the comorbidity rate far exceeds the rate of chance co-occurrence, indicating a close relationship between these two disorders (82). However, individuals with MDD show impairments in EFT and anticipated pleasure (83; 84). Lower ratings for detail/vividness, mental imagery, and personal significance uniquely predict lower state anticipatory pleasure (85). DD tends to be greater across a wide range of disorders, and depression that is associated with reduced optimism about the future could be a transdiagnostic indicator of this feature of DD. Therefore, if individuals with SUDs are not screened for other symptoms before undergoing EFT (EFT) interventions, the interventions are likely to be ineffective. Thus, the treatment of SUD is significantly more intricate and complex than anticipated, necessitating personalized interventions tailored to the unique characteristics of each individual.

Seventhly, accumulating neuroimaging evidence has provided important insights into the neural basis of EFT in healthy individuals. Episodic future thinking (EFT) is thought to reduce delay discounting by engaging a prospection network centered on the hippocampus and medial prefrontal cortex (mPFC) and by strengthening its interaction with valuation and control regions (41, 86). Through this network, EFT enhances the mental representation of future outcomes, making delayed rewards more vivid and salient. Activity in the hippocampus–mPFC system is functionally coupled with the ventral striatum, which encodes subjective reward value, and the anterior cingulate cortex (ACC), which supports cognitive control and goal regulation (41, 86, 87). This increased coupling allows future rewards to have a stronger impact on value computation, thereby shifting choices away from immediate rewards toward delayed ones. These network interactions offer a plausible neural basis through which EFT may influence DD by enhancing the representation and valuation of future outcomes. However, to date, no neuroimaging studies have directly examined neural correlates of temporal window expansion or construal level shifts as formal psychological constructs. Accordingly, future research may simultaneously assess neural activity, behavioral indices of EFT effect on DD, and explicit measures of temporal window and construal level representations in individuals with SUD. Establishing such mechanistic correspondence would not only advance theoretical models of EFT but also inform the development of mechanism-guided interventions for SUD, in which changes in DD are more likely to translate into meaningful and durable improvements in substance-related decision-making and clinical outcomes.

Finally, in addition to EFT, there may be additional novel strategies for improving intertemporal decision-making in individuals with SUD, such as working memory training and music-induced emotions (88, 89). According to the study by Zhao et al., working memory training, particularly the training of the updating component, can reduce impulsive decision-making in adolescents with low socioeconomic status levels. This type of training can decrease DD (i.e., the preference for immediate rewards) by enhancing the efficiency of working memory (88). Moreover, music therapy may serve as an effective non-pharmacological approach to modulate the emotional states of individuals with SUD by engaging with music that carries a spectrum of emotional resonance. This intervention has the potential to significantly impact decision-making processes. Specifically, the playback of joyful music may diminish the preference for immediate rewards, whereas the rendition of melancholic music may enhance such a preference (89). Therefore, in future studies, researchers may compare various methods for reducing impulsive decision-making in individuals with SUD, thereby improving our understanding of SUDs treatment options. For instance, it is worthwhile to explore whether positive autobiographical memory retrieval combined with music-induced emotions is a more effective intervention for individuals with SUD than either positive autobiographical memory retrieval or music-induced emotions alone.

### Article selection

The following search process was developed and implemented in the Web of Science database. The search used the following keywords: (“Episodic Future Thinking”, “Delay Discounting”, “Impulsivity”), (“Substance Use Disorder”, “Drug Use”, “Heroin”, “Alcohol”, “Opioids”, “Cannabis”, “Nicotine”, “Cocaine”, “Morphine”, “Amphetamines”, “Benzodiazepines”, “Phencyclidine” and “Methadone”), (“Abuse”, “Dependence”, “Use”, “Disorder”, “Addict”, “Misuse”).

A total of 684 potential papers were identified through the literature search. The full text of the selected articles was then extracted, read, and evaluated based on research criteria. The selection of studies was based on the following criteria: (1) full-text articles available (not conference abstracts); (2) the study was empirical rather than a review, commentary, or meta-analysis; (3) the study was not a case study; (4) the study measured the effects of EFT on DD in individuals with SUD. A final total of 21 articles was selected.
